# Management of Paediatric Cardiac Arrest due to Shockable Rhythm—A Simulation-Based Study at Children’s Hospitals in a German Federal State

**DOI:** 10.3390/children11070776

**Published:** 2024-06-27

**Authors:** Nadine Mand, Marieke Hoffmann, Anja Schwalb, Andreas Leonhardt, Martin Sassen, Tina Stibane, Rolf Felix Maier, Carolin Donath

**Affiliations:** 1 Neonatology and Paediatric Intensive Care, Department of Paediatrics, Philipps-University Marburg, 35043 Marburg, Germany; 2 Department of Paediatric Surgery, Philipps-University Marburg, 35037 Marburg, Germany; 3 Department of Child and Adolescent Psychiatry, Vitos Klinik, 34745 Herborn, Germany; 4 Department of Acute and Emergency Medicine, Diakonie-Hospital Wehrda, Philipps-University Marburg, 35041 Marburg, Germany; 5 Reinfried-Pohl-Zentrum for Medical Learning, Philipps-University Marburg, 35043 Marburg, Germany

**Keywords:** paediatric life support, paediatric cardiac arrest, shockable rhythm, simulation, simulation-based training

## Abstract

(1) Background: To improve the quality of emergency care for children, the Hessian Ministry for Social Affairs and Integration offered paediatric simulation-based training (SBT) for all children’s hospitals in Hesse. We investigated the quality of paediatric life support (PLS) in simulated paediatric resuscitations before and after SBT. (2) Methods: In 2017, a standardised, high-fidelity, two-day in-house SBT was conducted in 11 children’s hospitals. Before and after SBT, interprofessional teams participated in two study scenarios (PRE and POST) that followed the same clinical course of apnoea and cardiac arrest with a shockable rhythm. The quality of PLS was assessed using a performance evaluation checklist. (3) Results: 179 nurses and physicians participated, forming 47 PRE and 46 POST interprofessional teams. Ventilation was always initiated. Before SBT, chest compressions (CC) were initiated by 87%, and defibrillation by 60% of teams. After SBT, all teams initiated CC (*p* = 0.012), and 80% defibrillated the patient (*p* = 0.028). The time to initiate CC decreased significantly (PRE 123 ± 11 s, POST 76 ± 85 s, *p* = 0.030). (4) Conclusions: The quality of PLS in simulated paediatric cardiac arrests with shockable rhythm was poor in Hessian children’s hospitals and improved significantly after SBT. To improve children’s outcomes, SBT should be mandatory for paediatric staff and concentrate on the management of shockable rhythms.

## 1. Introduction

Paediatric cardiac arrest (CA) is a rare event both in and out of hospital (IHCA and OHCA). In Germany, an estimated 3000 to 4000 children are resuscitated each year, most of whom are already hospitalised at the time of CA [1,2]. OHCA occurs in approximately 3 per 100,000 children, with a quarter of these children being admitted to the hospital with ongoing cardiopulmonary resuscitation (CPR) [3]. Although mortality rates have decreased over the past 20 years, survival to discharge is as low as 17–40%, with slightly better outcomes for IHCA [4,5,6,7,8,9]. Outcomes are worse when paediatric CA occurs in the paediatric emergency department (PED) [10,11].

Respiratory and circulatory failure are the leading causes of paediatric CA, with asystole or pulseless electrical activity (PEA) being the first documented rhythm in most cases [12,13,14,15]. Shockable rhythms such as pulseless ventricular tachycardia (pVT) and ventricular fibrillation (VF) are initially seen in approximately 5–15% of paediatric CA [3,5,14,16]. Although patient outcomes appear to be better when the initial rhythm is shockable [17,18,19], paediatricians are uncomfortable using defibrillators and errors are common [20,21].

Studies investigating the quality of paediatric resuscitation have shown considerable deficiencies [20,22,23]. Paediatricians feel unsure and unprepared when providing CPR [24,25,26]. However, both neurological outcome and survival depend significantly on the time of initiation and the quality of care provided [27,28,29]. For adult and neonatal resuscitations, it has been shown repeatedly that close adherence to the resuscitation guidelines is a key factor in improving the patient’s outcome [27,30]. The data regarding paediatric patients after the neonatal period is limited [31].

Simulation-based training (SBT) can improve team performance, procedural skills, and guideline adherence in medical students, nurses, and physicians [32,33,34,35], and improves the quality and timely implementation of time-critical measures during resuscitation [36,37,38]. In an effort to improve patient safety in Hessian hospitals, the Hessian Ministry for Social Affairs and Integration (HSMI) offered a standardised two-day paediatric SBT for all Hessian children’s hospitals. Hesse is a federal state in Germany with a total population of 6.3 million. Fifteen children’s hospitals, including three university hospitals, provide emergency and inpatient care for a population of 1.1 million children and adolescents under the age of 18 [39]. In this multicentre study, we aimed to investigate the quality of paediatric life support (PLS) in simulated paediatric CA due to a shockable rhythm in Hessian children’s hospitals, and the effect of SBT on the care provided. Secondly, we evaluated the timing of medication administration and the frequency of medication errors before and after SBT.

## 2. Materials and Methods

### 2.1. Study Setting

Between April 2017 and January 2018, paediatric SBT was conducted in 11 of the 15 children’s hospitals in Hesse, Germany. Although each of these hospitals provides emergency care for critically ill children, there is a high variability in patient capacities and annual patient volume (see Table 1). Only 5 of these 11 children’s hospitals maintain a paediatric intensive care unit (PICU). 

Ethical approval was obtained from the Ethics Committee of the Philipps-University of Marburg (AZ: 172/16). Written informed consent was obtained from all study participants.

### 2.2. Simulation-Based Training 

**Figure 1:**
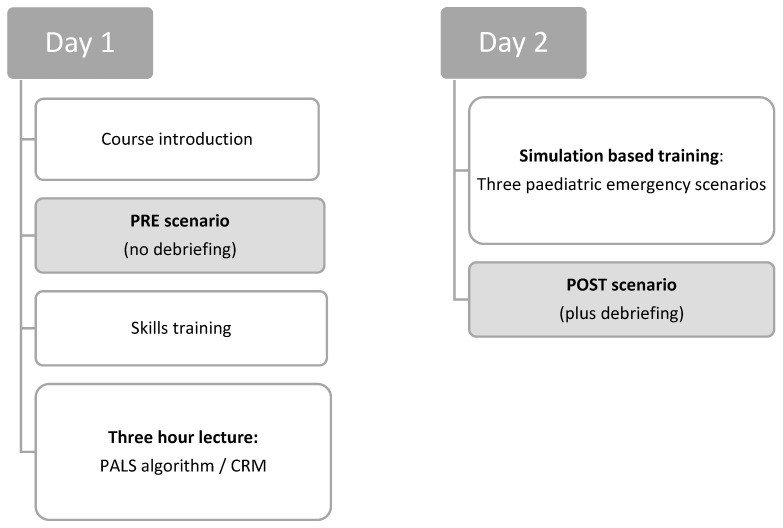
Study design.

SBT was standardised for all children’s hospitals. Each hospital could enrol a maximum of 20 participants in the training. SBT was delivered as an in-house training on two consecutive days and consisted of a 3-h interactive lecture and three simulation scenarios (see Figure 1). Two hours of the lecture focused on the recognition of critically ill children, and paediatric basic and advanced life support (PBLS and PALS), including cardiac rhythm recognition and shockable and non-shockable cardiac rhythm algorithms. Crew resource management (CRM) aspects were covered in the third hour. Simulation scenarios were performed with high-fidelity mannequins (a Gaumard HAL3010 tetherless newborn simulator and a HAL3005 tetherless 5-year-old paediatric simulator). These scenarios consisted of a respiratory, a circulatory, and a neurological paediatric emergency leading to apnoea and cardiac arrest with a non-shockable cardiac rhythm. Varying resuscitation teams of up to six participants took part in these simulation scenarios. Each scenario was followed by a structured debriefing. 

Depending on the hospital’s preference, the training was conducted in PEDs, on inpatient wards, or PICUs, using the emergency medical equipment available on site. 

The course was developed by experts in paediatric emergency medicine, paediatric intensive care, and simulation-based training. It was piloted in the PICU of the Department of Paediatrics at Philipps-University in Marburg, Germany. All instructors were formally trained as simulator instructors and EPALS providers.

A total of 188 specialized paediatric nurses and physicians participated in this SBT. 

### 2.3. Study Participants

Study participants were recruited from the SBT participants at each children’s hospital and included paediatric nurses and physicians with different levels of experience. Participation was voluntary. Study participants formed study teams independently, with four study participants each, including at least one nurse and one physician. Team composition differed in each simulation and study scenario.

Each study participant completed questionnaires about demographics and previous resuscitation experience.

### 2.4. Study Scenarios

Immediately before and after the SBT, study participants took part in two study scenarios (PRE and POST scenario, see Figure 1), which were recorded using an audio-video system. The PRE and POST scenarios differed in the patient history provided to the teams but followed the same clinical progression of apnoea and cardiac arrest with identical vital signs. 

Study scenarios were scripted to last 12 min regardless of the actions performed. A critically ill infant was presented to the study teams in the PED or on the paediatric ward. After two minutes, the patient went into apnoea and CA with a shockable cardiac rhythm. Eight minutes later, the patient had a return of spontaneous circulation (ROSC) regardless of the study teams’ resuscitation interventions. ROSC could have been achieved earlier if the study teams performed the PALS algorithm correctly (adequate CPR technique, three correctly dosed shocks, epinephrine, and amiodarone at the correct dose and time). The scenario was terminated two minutes after ROSC. Study participants were not debriefed after the PRE scenarios.

### 2.5. Performance Evaluation

Guideline adherence was assessed using a performance evaluation checklist (PEC) based on a PEC already validated in German paediatric emergency teams [40]. The development of the performance evaluation checklist on shockable rhythms used in this study is described elsewhere [41].

This PEC consists of 31 items, which are divided into three evaluation categories (task not performed; task performed partially, incorrectly, or with delay; and task performed completely). Each item is weighted between 1 and 5 according to its importance to treatment success (see Supplementary Materials). Thus, the PEC illustrates the complexity of a resuscitation situation. To enable the most accurate evaluation of all items, a rater training handbook was developed, in which the rating of each item was specified [41]. A maximum of 284 points could be achieved, and a minimum of 142 points were needed for the team’s performance to be rated a sufficient resuscitation. Additionally, the times to initiation of bag-mask ventilation (BMV), chest compressions (CC), and defibrillation were recorded. 

Study scenarios were retrospectively analysed by specially trained raters following a rater training programme achieving good interrater reliability (Kendall’s tau > 0.7) [41].

### 2.6. Data Analysis

Data were analysed using IBM SPSS Statistics Version 29.0. Categorical variables are expressed as frequencies and percentages. Chi-squared tests were used for comparing frequencies of categorical variables in PRE and POST scenarios. Arithmetic mean and standard deviation were used for characterizing initiating times (e.g., time to ventilate) and unpaired *t*-tests for comparing PRE and POST scenarios. The level of significance was set at *p* < 0.05.

## 3. Results

In total, 179 of 188 (95%) participants agreed to take part in the study and formed 51 PRE and 47 POST scenario study teams. After the exclusion of five videos due to poor audio quality, 47 PRE and 46 POST scenario videos were analysed (see Table 1). The participants’ professional roles and previous clinical experience are described in Table 2.

### 3.1. Paediatric Basic Life Support

**Figure 2:**
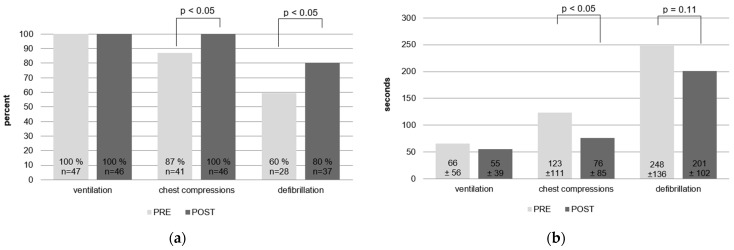
(**a**) Frequency of life support measures before and after SBT; (**b**) time to initiate life support measures before and after SBT.

Ventilation was initiated by all teams in PRE and POST scenarios, and in 93% as BMV. There was no significant reduction in initiation time (PRE 66 s ± 56 s, CI95 49–82 s; POST 55 s ± 39 s, CI95 43–66 s; n.s.) (see Figure 2a,b). 

Before SBT, 87% resuscitation teams recognized CA and started CC, which improved significantly to 100% after SBT (*p* = 0.012). The time to initiate CC significantly declined after SBT (PRE 123 ± 111 s, CI95 88–157 s.; POST 76 ± 85 s, CI95 51–101 s; *p* = 0.030).

The number of teams initiating ventilation and CC within 2 min after CA increased from 51% to 80% (*p* < 0.01). There was no significant change in the number of teams initiating CPR within one minute after CA (PRE 30% vs. POST 46%, *p* = 0.11).

### 3.2. Paediatric Advanced Life Support

Of the PRE resuscitation teams, 13% checked and recognised the rhythm after CA; this did not improve significantly, with 22% of POST resuscitation teams recognising and verbalising the correct rhythm after SBT (*p* = 0.09). Slightly more teams started preparing to defibrillate after SBT (PRE 75% vs. 89%, *p* = 0.067), while the number of teams that defibrillated increased significantly from 60% to 80% (*p* = 0.028). Time to defibrillation did not decrease significantly, from PRE 248 s ± 136 s (min. 31 s, max. 510 s) to POST 201 s ± 102 s (min. 34 s, max. 508 s, *p* = 0.11).

Reversible causes of CA (“4H4T”) were at least partially checked by 34% of PRE resuscitation teams; this improved significantly after SBT, with 54% of POST resuscitation teams checking (*p* = 0.049). No team systematically checked all reversible causes. 

The overall score achievable in the PEC improved significantly, from PRE 91 ± 26 points (CI95 83–99) to POST 118 ± 28 points (CI95 109–126, *p* < 0.001). While none of the teams achieved the minimum of 142 points indicating a sufficient resuscitation before SBT, 12 (26%) resuscitation teams reached that goal after SBT (*p* < 0.001).

### 3.3. Medication during Resuscitation

Medication was rarely used according to PLS guidelines in the PRE scenarios, but this improved significantly after SBT. 

Epinephrine was applied at the correct time (after the 3rd shock) with the correct dose (10 µg/kg) in no PRE and 9% of POST resuscitation teams (*p* < 0.001). Average doses used improved from PRE 26 ± 55 µg/kg (CI95 0.7–50.8) to POST 10 ± 1 µg/kg (CI95 9.8–10.3), though not significantly (*p* = 0.08). There were two dosing errors in the PRE resuscitation teams, with epinephrine doses of 100 µg/kg and 250 µg/kg. No dosing error occurred after SBT (*p* = 0.059). 

Amiodarone was used with the correct dose (5 mg/kg) in 9% of PRE and 33% of POST resuscitation teams (*p* < 0.001), though it was never used at the correct time (after the 3rd shock). Average doses were 5 mg/kg (PRE 5 ± 0 mg/kg, POST 5 ± 0.2 mg/kg, n.s.), and no dosing errors occurred.

## 4. Discussion

We analysed the quality of paediatric life support in simulated paediatric cardiac arrests due to shockable rhythm and the effect simulation-based training had on the care provided. In total, 179 nurses and physicians from 11 children’s hospitals participated, forming 47 study resuscitation teams. To the best of our knowledge, this is the first multicentre study to systematically investigate the care of paediatric emergencies in children’s hospitals in a German federal state. 

We found great uncertainty in recognising CA and initiating basic life support amongst the health care professionals before SBT. Although all teams recognised the need for ventilation, which was initiated on average within one minute, ventilation of a pulseless patient is not effective without supporting circulation. Only 30% of the teams started ventilation and chest compressions within one minute, and only 51% of the teams within two minutes after the onset of CA. Moreover, 13% of the teams did not start CC at all, which would lead to a fatal outcome for those patients. However, delaying CC in a pulseless patient also leads to adverse outcomes, including a lower rate of ROSC [29]. In our study, CC were started on average two minutes after CA. Similar delays have been reported in other studies [20,42,43], with participants performing unnecessary actions before initiation of CC due to incorrect prioritization [43], or CC were not initiated at all [43]. Resuscitation experience and frequency of resuscitation trainings seem to be crucial for correct and prompt algorithm adherence [43]. However, retention of basic life support skills among paediatricians has been reported to be poor [44,45], and the quality of CPR is significantly better when training was very recent [46]. Only half of our study participants had previous experience of CPR and had received specific training within the previous 12 months. This may explain the low rate and late initiation of CC in our cohort. In addition, the quality and extent of previous training was not assessed, which may have contributed to the weak performance.

Adherence to paediatric advanced life support measures was even more discouraging. Before SBT, only six teams checked and verbalised the shockable cardiac rhythm in our simulated patient, even though early differentiation between shockable and non-shockable rhythms is emphasised in resuscitation guidelines [47]. If in doubt, the rhythm is considered shockable and early defibrillation is recommended [47]. Patients may transition between cardiac rhythms during resuscitation [5,12,48], with less favourable outcomes in patients with secondary development of a shockable rhythm [48,49]. Though defibrillators were ordered in 75% of the study scenarios at one point, only 60% of our study teams used them on their patients. On average, defibrillation was performed more than 4 min after CA. Hunt et al. found similar delays in defibrillation, which they attributed to a lack of experience with previous usage on a human or mannequin [20]. However, contrary to adult data on shockable rhythms [50], time to defibrillation was not associated with decreased survival in a large cohort of 477 paediatric patients with a pulseless shockable rhythm, but all patients received defibrillation at some point [51]. In our cohort, 40% of patients were not defibrillated, which would have resulted in a fatal outcome for these patients.

Simulation-based training significantly improved the quality of resuscitation. All teams recognised CA in their simulated patients and initiated CC in almost half the time compared to before SBT. Paediatric advanced life support also improved, with significantly more teams recognising the correct rhythm and defibrillating the patient more often and almost a minute faster. In general, team performance and adherence to guidelines improved significantly after our standardized SBT. While none of the teams achieved the minimum of points in the performance evaluation checklist before SBT, one out of four resuscitation teams reached that goal after SBT (*p* < 0.001). Though encouraging, similar studies have shown better results. In a multicentre study by Gilfoy et al., only one of sixty (2%) resuscitation study teams did not defibrillate after SBT, compared to nine teams (15%) before SBT [35]. In contrast to our study, Gilfoy et al. only evaluated resuscitation teams from university children’s hospitals with similar patient volumes, which may explain the better initial data. As other studies have pointed out, guideline adherence is dependent on paediatric patient volumes [37]. Participants in our study originated from PEDs, PICUs, and inpatient care. The high variability in experience among participants may have been the reason why, even after SBT, 20% of our study teams still failed to defibrillate. Since paediatric emergencies do not exclusively occur in PICUs, with a worse outcome in inpatient wards and PED [11,52], it is crucial to train all paediatric health care professionals. 

SBT also had an effect on medication usage and medication errors. Epinephrin usage was high, with 43% of PRE teams and 78% of POST teams using it at some point. However, correct timing was rare and is potentially harmful; this improved significantly after SBT (*p* < 0.001). Studies on real-life adult CA due to shockable rhythm found that one in five patients received epinephrine before defibrillation, resulting in higher morbidity and mortality [53,54]. Amiodarone is recommended after the third shock in shockable rhythm [55]. Though there were no dosage errors, resuscitation teams used it rarely. 

Overall, SBT significantly improved paediatric basic and advanced life support in our cohort. Adherence to resuscitation guidelines improves patient outcomes [47], so the changes seen here, especially in paediatric basic life support, suggest an immense impact on the real-life care of children with CA in the children’s hospitals studied.

### Strengths and Limitations

We systematically investigated paediatric life support in German children’s hospitals in a defined federal state. With healthcare professionals from 11 different children’s hospitals participating, almost 75% of inpatient care in Hesse was represented. The interprofessional team composition and in-house simulation using locally available emergency medical equipment reflected the reality of paediatric emergency care. Resuscitation teams varied during the SBT, as well as during the study scenarios, so the effects described here cannot be attributed to familiarity with working in a particular team. Participants were recruited from PEDs, PICUs, and general paediatric wards, resulting in a wide range of previous experience and expertise. This high variability among participants may have been the reason why SBT did not lead to significant improvements in all items investigated. Since paediatric emergencies do not exclusively occur in PICUs, with a worse outcome in inpatient wards and PEDs [11,52], it is crucial to train all paediatric health care professionals. 

Our study design might also have influenced the results. As the management of shockable rhythms was taught only theoretically, with simulation scenarios focusing on non-shockable rhythms, there was no familiarization with the defibrillator. Another simulation study found that up to 80% of paediatricians had difficulty using the defibrillator, which led to inappropriate shock administration [20]. A multicentre analysis of more than 400 shocks delivered during 159 real-life paediatric CA events showed that although 88% of events occurred in a PICU or PED, with only 12% in other hospital settings such as operating theatres or inpatient wards, inappropriate shock delivery was similarly high, at 30% in PICUs and PEDs and 27% in other hospital settings [21]. Future studies need to focus on paediatric healthcare professionals using the correct algorithms for shockable rhythm in paediatric CA.

Finally, we did not evaluate which aspects of our SBT specifically led to the observed changes in PLS and how long these effects lasted. As proposed by simulation networks, SBT should be of high quality to provide standardised learning conditions for trainees and ensure lasting effects [56]. The proposed framework of simulation trainer qualifications, effective learning and simulation environments, and the process of scenario development and implementation, including standardised debriefing [56], was met in our SBT. The exact frequency with which it should be applied has not yet been defined. 

## 5. Conclusions

To the best of our knowledge, this is the first multicentre study to systematically investigate the care of paediatric emergencies in children’s hospitals in a German federal state. We were able to demonstrate an improvement in the management of paediatric CA due to shockable rhythm after structured simulation-based training. Further training and research should focus on rhythm checks and the correct choice of paediatric advanced life support measures. 

## Figures and Tables

**Table 1:** Characteristics of study sites and analysed study scenarios.

Study Site	Hospitals *n/N* (%)	PRE Scenarios *n/N* (%)	POST Scenarios *n/N* (%)
Small children’s hospital (≤50 beds)	3/11 (27)	14/47 (30)	12/46 (26)
Medium children’s hospital (51–100 beds)	4/11 (37)	18/47 (38)	19/46 (41)
Large children’s hospital (>100 beds)	2/11 (18)	6/47 (13)	7/46 (15)
University hospital	2/11 (18)	9/47 (19)	8/46 (18)

**Table 2:** Characteristics of the study participants.

Professional and Educational Characteristics	*n* (%) of Cohort (*N* = 179)
Professional role	
Head of department	1 (0.6)
Senior physician	16 (8.9)
Resident physician	65 (36.3)
ICU nurses	20 (11.2)
Nurses	72 (40.2)
n/a	5 (2.8)
Years of experience ^1^	
Senior physician	6.7 ^2^ (±6.2) years
Resident physician	3.4 (±3.8) years
ICU nurses	11.7 (±7.8) years
Nurses	17.5 (±13.0) years
Previous experience with paediatric resuscitations ^3^	77 (43.0)
Previous resuscitation training	155/179 (86.6)
Within the last 12 months	88/155 (56.8)
Previous crew resource management training	21/179 (11.7)
Within the last 12 months	14/21 (66.7)

^1^ Refers to the number of years worked in the current role; ^2^ arithmetic mean (±standard deviation); ^3^ neonatal resuscitations were explicitly excluded.

## Data Availability

The data presented in this study are available on request from the corresponding author due to ethical reasons.

## Supplementary Materials

The following supporting information can be downloaded at: https://www.mdpi.com/article/10.3390/children11070776/s1, Performance Evaluation Checklist (PEC).
