# Supplementary material for: Management of Paediatric Cardiac Arrest due to Shockable Rhythm—A Simulation-Based Study at Children’s Hospitals in a German Federal State
Source: Children (Basel). 2024 Jun 27;11(7):776. doi: 10.3390/children11070776 (PMC11274526; doi:10.3390/children11070776)
Supplement: Supplementary file 1 [file children-11-00776-s001.zip › children-3045447-supplementary.pdf]

PEC-PVT

					Time	Weighting
**1**	**General tasks and evaluation**	**0 point** *Not done*	**1 point** *Partially OR incorrectly done OR not done in a timely manner*	**2 points** *Done correctly, completely AND in a timely manner*	**Time**	**Weighting**
1-1	Put on gloves	□ ø	□ not all OR late	□ everyone AND in time	3 min	2
1-2	Equipment check	□ ø	□ partially OR late	□ completely AND in time		4
1-3	Connect monitors	□ ø	□ partially OR late	□ completely AND in time		4.5
1-4	Call for help	□ ø	□ late OR after recommendation of instructor	□ in time		4.5
1-5	ABC-evaluation	□ ø	□ partially OR late	□ completely AND in time		5
1-6	Oxygen	□ ø	□ nasal cannula OR blow by oxygen	□ 100% oxygen applied		5
1-7	Inform team members about diagnosis	□ ø	□ unspecific diagnosis OR late	□ specific AND in time		5
**2**	**Evaluation and treatment AFTER apnea and cardiac arrest**	**0 point**	**1 point**	**2 points**		**W**
2-1	Assess airway and breathing	□ ø	□ > 30 sec OR no verbalisation	□ ≤ 30 sec AND verbalisation		5
2-2	Begin bag-mask-ventilation	□ ø	□ > 30 sec	□ ≤ 30 sec		5
2-3	Optimize ventilation if necessary	□ ø	□ > 30 sec OR no verbalisation	□ ≤ 30 sec after beginn of ventilation AND verbalisation AND optimisation if necessary		5
2-4	Check pulse	□ ø	□ > 30 sec OR no verbalisation	□ ≤ 30 sec AND verbalised		5
2-5	ECG rhythm check	□ ø	□ rhythm check BUT no verbalisation	□ correct rhythm verbalised		4.5
2-6	Start CPR	□ ø	□ > 30 sec after cardiac arrest OR incorrect CPR technique	□ ≤ 30 sec after cardiac arrest AND good CPR technique		5
2-7	Prepare defibrillation	□ ø	□ > 30 sec after cardiac arrest	□ ≤ 30 sec after cardiac arrest		5
2-8	Establish IV/IO infusion	□ ø	□ > 120 sec after cardiac arrest	□ ≤ 120 sec after cardiac arrest		4.5
2-9	Defibrillation (first)	□ ø	□ > 90 sec after cardiac arrest OR incorrect dose OR incorrect mode	□ ≤ 90 sec after cardiac arrest AND correct dose AND correct mode		5
2-10	CPR (continuous)	□ ø	□ > 10 sec after 1st shock OR incorrect CPR technique	□ ≤ 10 sec after 1st shock AND good CPR technique		5
2-11	ECG rhythm check	□ ø	□ rhythm check BUT no verbalisation OR < 100 sec / > 120 sec after 1st shock	□ correct rhythm verbalised AND between 100 and 120 sec after 1st shock		5
2-12	Defibrillation (second)	□ ø	□ < 100 sec OR > 120 sec after 1st shock OR incorrect dose OR incorrect mode	□ between 100 and 120 sec after 1st shock OR after 10 CPR cycles AND correct dose AND correct mode		5
2-13	CPR (continuous)	□ ø	□ > 10 sec after 2nd shock OR incorrect CPR technique	□ ≤ 10 sec after 2nd shock AND good CPR technique		5
2-14	ECG rhythm check	□ ø	□ rhythm check BUT no verbalisation OR < 100 sec / > 120 sec after 2nd defibrillation	□ correct rhythm verbalised AND between 100 and 120 sec after 2nd shock		5
2-15	Defibrillation (third)	□ ø	□ < 100 sec OR > 120 sec after 2nd shock OR incorrect dose OR incorrect mode	□ correct rhythm verbalised AND between 100 and 120 sec after 2nd shock		5
2-16	CPR (continuous)	□ ø	□ > 10 sec after 3rd shock OR incorrect CPR technique	□ ≤ 10 sec after 3rd shock AND good CPR technique		5
2-17	ECG rhythm check	□ ø	□ > 10 sec after 3rd (or last) shock OR incorrect CPR technique	□ correct rhythm (sinus rhythm) verbalised AND between 100 and 120 sec after 3rd (or last) shock		5
2-18	Check pulse	□ ø	□ pulse check but no verbalisation OR < 100 sec / > 120 sec after 3rd (or last) shock	□ pulse check verbalised AND between 100 und 120 sec after 3rd (or last) shock		5
2-19	Epinephrine	□ ø	□ wrong dose OR given before 3rd shock	□ IV / IO correct dose AND correct time		5
2-20	Amiodarone	□ ø	□ wrong dose OR given before 3rd shock	□ IV / IO correct dose AND correct time		4
2-21	Assess blood pressure OR recap time	□ ø	□	□ after return of spontaneous circ.		3
**3**	**Search for reversible causes**	**0 point**	**1 point**	**2 points**		**W**
3-1	4 Hs & HITS-evaluation	□ ø	□ incomplete	□ complete		4.5
3-2	Blood withdrawal for blood gas analysis, glucose and electrolyte	□ ø	□ done but no verbalisation	□ done AND results verbalised		3.5
3-3	Early planning of other treatments	□ ø	□	□ done		3

**Time to 1st shock:** **sec**	**Time to CPR:** **sec**	**Time to ventilation:** **sec**	**Weight:**
**Dose 1st / 2nd / 3rd shock:**	**Epinephrine dose:** **µg**	**Amiodarone dose:** **mg**	**kg**
